# Quality of life and its associated factors among adult PLWHA on ART in Fako Division, South West Region of Cameroon

**DOI:** 10.1371/journal.pmen.0000697

**Published:** 2026-10-26

**Authors:** Bombey Mary-Flavie Nkanjo, Ayuk Betrand Tambe, Achidi Aduni Ufuan, Sam Denise Movuh, Tendongfor Nicholas

**Affiliations:** 1 Department of Public Health and Hygiene, Faculty of Health Sciences, University of Buea, Buea, Cameroon; 2 Division Human Nutrition, Faculty of Medicine and Health Sciences, Stellenbosch University, Cape Town, South Africa; 3 Department of Biochemistry, Faculty of Science, University of Buea, Buea, Cameroon; 4 Plan International, Yaoundé, Cameroon; Macao Polytechnic University, MACAO

## Abstract

Human immunodeficiency virus and acquired immunodeficiency syndrome (HIV/AIDS) remain a major global health challenge. This study assessed the quality of life and its associated factors among people living with human immunodeficiency virus and acquired immunodeficiency syndrome (PLWHA) on antiretroviral therapy in two treatment centers in the South West Region of Cameroon. A cross-sectional study was conducted among 464 PLWHA on ART at two treatment centers in the Fako Division, Southwest Region of Cameroon, from June 2024 to August 2024. Quality of life was assessed using the World Health Organization’s Quality of Life brief questionnaire (WHOQOL-BREF). Data was analyzed using the statistical package for the social sciences version 25.0. Statistical significance was set at a p-value <0.05 at a 95% confidence interval. Participants’ mean age was 48.8 ± 11.7 years and 79.5% were female. Most had been on ART for ≥3 years (92.2%) and were virally undetectable (85.8%). Mean domain scores were highest for physical health (72.93 ± 12.79) and psychological health (70.55 ± 13.35), and lowest for social relationships (62.03 ± 16.83) and environmental health (64.24 ± 11.08). The mean overall QOL score was 269.75 ± 42.58. In multivariable analysis, lower overall QOL scores was associated with older age, primary education, and monthly income <100 USD, while higher overall QOL scores was associated with being married/divorced, absence of HIV-related symptoms, and absence of depression. QOL among PLWHA on ART in Fako Division was moderate-to-good, with the social and environmental domains most affected. Socioeconomic vulnerability, aging, depressive symptoms, and clinical symptom burden were key determinants, underscoring the need to integrate mental health screening and support, strengthen symptom management, and address socioeconomic barriers within HIV care.

## Background

HIV/AIDS continues to pose a significant global health challenge, with an estimated 39.9 million people living with the virus worldwide as of 2023 [1]. Despite tremendous advancements in knowledge, care, and prevention, HIV/AIDS still has a major influence on public and individual health, especially in sub-Saharan Africa, where it is most prevalent [2]. In Cameroon, the prevalence of HIV is approximately 2.7%, with an estimated 450,000 people living with HIV/AIDS (PLWHA) [3]. The burden of HIV/AIDS is not only evident in the number of infections but also its profound impact on the QOL of affected individuals. The chronic nature of HIV and its accompanying effects are highlighted by the World Health Organization’s (WHO) estimation that a considerable percentage of the Disability-Adjusted Life Years (DALYs) lost worldwide are caused by HIV-related illnesses [4].

WHO has defined QOL as “individuals’ perceptions of their position in life in the context of the culture and value systems in which they live and in relation to their goals, standards, expectations, and concerns”, according to this definition, QOL is a subjective assessment that is influenced by social, cultural, and environmental factors [5]. Studies have shown that PLWHA often experience reduced QOL due to various factors, including the stigma associated with the disease, the side effects of antiretroviral therapy (ART), and co-morbidities. Poor QOL can adversely affect adherence to ART, which is important for maintaining viral suppression and overall health [6].

Furthermore, QOL is influenced by socio-economic status, mental health, and the availability of social support, all of which are critical in managing the disease and ensuring better health outcomes [7]. The factors associated with QOL among PLWHA vary, from socio-demographic variables, such as age, gender, marital status, and education. The factors have been shown to significantly impact the QOL of individuals living with HIV [8]. Additionally, mental health conditions, particularly depression and anxiety, can substantially diminish QOL and complicate the management of HIV [9]. Furthermore, access to healthcare services and the quality of care received also play a vital role in determining QOL, as these factors influence both physical health and emotional well-being [7]. Moreover, the support from family, friends, and community networks is essential for enhancing the QOL of PLWHA by providing emotional and practical assistance [7].

Limited research has been done on the quality of life among PLWHA in Cameroon. This is of utmost concern as a better understanding of the factors that influence QOL is important for developing targeted interventions to improve the lives of these individuals. This study aims to assess the QOL and its associated factors among PLWHA on ART in two treatment centers in Fako Division.

## Materials and methods

### Ethics statement

This study was approved by the Institutional Review Board of the Faculty of Health Sciences, University of Buea (Ref. No. 2024/2459-03/UB/SG/IRB/FHS). Administrative clearance was obtained from the Regional Delegation of Public Health for the South West region (Ref. No P42/MINSANTE/SWR/RDPH/CB.PT/680/512), and written informed consent was obtained from the patients recruited into this study. Potential participants were informed before the data collection that all conversations and data derived from the study was kept strictly confidential. All research was performed in accordance with relevant guidelines/regulations. All reports and publications did not carry any identifier that could be linked to participants to ensure anonymity.

### Study design

This study adopted a hospital-based cross-sectional design. Firstly, it allows for efficient assessment of quality of life at a single time point among a large, diverse population of PLWHA, providing a comprehensive snapshot of current QOL status under existing treatment programs. Secondly, the design is particularly suitable for QOL research as it captures patient-reported outcomes that reflect cumulative treatment effects without the resource intensity of longitudinal studies.

### Study site and population

The study was carried out in Buea and Limbe Regional Hospitals of the South West Region of Cameroon. Buea and Limbe Regional Hospitals serve as primary HIV treatment centers for the South West Region, which captures patients from both urban (Limbe - coastal commercial hub) and semi-urban (Buea - administrative capital) settings, ensuring diverse socioeconomic and cultural representation. The facilities offer comprehensive HIV services, including viral load monitoring, CD4 testing, and standardized ART protocols, ensuring reliable clinical data collection and uniform care standards for comparison. These hospitals serve as secondary referral centers for primary healthcare facilities, private clinics, and faith-based organizations throughout the region, capturing patients who may have initiated care elsewhere but require specialized services.

The study population consisted of adult persons living with HIV/AIDS aged 18–78 years attending the HIV care and treatment centre for routine health education and drug refills. Included in the study were PLWHA with a history of at least six months of antiretroviral therapy (ART) at the treatment centre. Excluded from the study were those who were critically ill, and pregnant women.

### Sampling

The sample size was determined using the single population formula N = (Z_α/2_)^2^ s^2^/ d^2^ [10]^.^ Where the Z-value of 1.96 for a 95% confidence level, s is the standard of deviation of 0.53 for the overall quality of life score based on previous literature [11], and a margin of error of 5%. This yielded an initial sample size of 432. To account for a possible non-response rate of 10%, the final sample size was adjusted to 480 participants. A total of 464 participants were recruited consecutively as they consented during follow-ups.

Participants were consecutively recruited as they visited the treatment centers for drug refill. This approach was used because participants return to treatment centers to refill their stock of medication at different intervals, and more too, these two hospitals are reference hospitals with the majority of HIV/AIDS patients being enrolled for treatment.

### Study variables

The dependent variable in this study was QOL among PLWHA. This variable was determined using the WHOQOL BREF tool [12]. The independent variables included the sociodemographic, clinical and lifestyle characteristics of the participants.

### Procedures

Data were obtained using a structured and pretested questionnaire based on the WHOQOL- BREF instrument. The questionnaire was sectioned into three parts: sociodemographic information, clinical information, and quality of life. The WHOQOL-BREF instrument was adopted to assess the respondents’ quality of life. This is an international, cross-culturally comparable QOL assessment instrument [13]. The WHOQOL-BREF instrument comprises 26 items, which measure physical health, psychological health, social relationships, and the environment [14]. The WHOQOL-BREF was selected because it is a widely validated instrument for assessing general quality of life across different populations and health conditions [15]. Its use enables comparison of quality-of-life outcomes between people living with HIV and the general population. The instrument is also shorter and less burdensome for respondents than disease-specific tools, making it appropriate for use in clinical settings [16] and its availability in English, which is the official language of the study population. The tool has been widely used in other settings in Cameroon [5,17,18].

The equation recommended by WHO was used to calculate the total score of each domain. Because the number of items varies across the four domains, the score of each domain is calculated by multiplying the average score of all items in the domain by the same factor of 4. With each item’s score ranging from 1 to 5, the total score of each domain will be between 4 and 20, which was transformed to scores ranging from 0 to 100 (a higher score reflecting better QOL) [19]. The higher the score, the better the QOL, which makes it convenient to compare the scores in different domains, populations, and studies. Besides, the WHOQOL-BREF is a self-administered questionnaire and gives the evaluation of the subject’s QOL in the past four weeks [12]. In order to obtain the overall quality of life score, the scores of the four domains are summed. Persons accessing ART at the hospital were approached after they had received their service, and the purpose of the study was explained to them. Upon completion of the study participation process and assurance of ethical concerns and all other concerns addressed, respondents then completed the self-administered questionnaire, which assessed their perceived quality of life over the previous four weeks. Two trained research assistants were present to supervise the data collection process and provide clarification where necessary between June and August 2024. For participants who were unable to read or fully understand the questionnaire, the research assistants assisted by reading and explaining the questions to ensure accurate completion.

### Validity of the study

Validity is the accuracy and meaningfulness of inferences that are based on research results and how accurately the data obtained represent the variable of the study [20]. The overall quality of life scale demonstrated acceptable internal consistency (Cronbach’s α = 0.783). The physical (α = 0.719) and psychological (α = 0.755) domains showed satisfactory reliability, whereas the environmental domain (α = 0.575) was moderate and the social domain (α = 0.340) indicated poor internal consistency.

### Data analysis

Data was cleaned in Microsoft Excel before being imported into SPSS software for statistical analysis. The data were exported to Statistical Package for Social Sciences (SPSS) software, version 27, for analysis. Frequencies, proportions, percentages, mean and standard deviation were used for descriptive analysis. The simple linear regression was used for the bivariate analysis and the multivariate linear regression was used for the multivariate analysis. Variables with p < 0.2 in the bivariate analysis entered the multivariate model. Variables with P < 0.05 in the multivariate model was considered statistically significant. For the quality of life, scores of each domain were transformed into a scale of 100 [21]. The overall QOL score was then computed by summing the total scores of the four transformed domains [22].

## Results

### Demographic characteristics of PLWHA in Buea and Limbe Regional Hospitals

The socio-demographic characteristics of the study participants are presented in Table 1. A total of 464 persons living with HIV/AIDS on ART were recruited into the study. The age of participants ranged from 18 to 78 years, with a mean of 48.80 ± 11.67 years. Less than half of the population (43.5%) were between the ages of 34–49 years. More than three-quarters (79.5%) were females (79.5%), and 20.5% were males. Less than half (46.3%) had a primary level of education.

**Table 1:** Socio-demographic characteristics of PLWHA in Buea and Limbe Regional Hospitals.

Variable	Categories	Frequency	Percentage
Age group (Years)	18-33	44	9.5
	34-49	202	43.5
	50-65	185	39.9
	>65	33	7.1
	**Total**	**464**	**100**
Gender	Female	369	79.5
	Male	95	20.5
	**Total**	**464**	**100**
Marital status	Divorced	12	2.6
	Married	197	42.5
	Single	158	34.1
	Widow(er)	97	20.9
	**Total**	**464**	**100**
Religion	Christian	458	98.7
	Muslim	6	1.3
	**Total**	**464**	**100**
Level of education	None	13	2.8
	Primary	215	46.3
	Secondary	182	39.2
	Tertiary	54	11.6
	**Total**	**464**	**100**
Average income (USD)	< 100	198	42.6
	100-200	160	34.5
	201-400	49	10.6
	> 400	57	12.3
	**Total**	**464**	**100**
Location	Regional Hospital Buea	217	46.8
	Regional Hospital Limbe	247	53.2
	**Total**	**464**	**100**

### Clinical and lifestyle characteristics of PLWHA in Buea and Limbe Regional Hospitals

Table 2 shows the clinical and lifestyle characteristics of the study participants. The majority of the participants (92.2%) had been on ART for 3 years or above. The majority (93.8%) of them did not have HIV related symptoms, and were on a first-line regimen (86.9%). More than half of the participants consumed alcohol (63.4%), and most (97.4%) were not smokers.

**Table 2:** Clinical and lifestyle characteristics of PLWHA in Buea and Limbe Regional Hospital.

Variable	Categories	Frequency (n)	Percentage (%)
Duration on ART	3 years and above	428	92.2
	6 months to 3 years	36	7.8
	**Total**	**464**	**100**
Clinical stage of HIV/AIDS	Stage I	243	52.4
	Stage II	84	18.1
	Stage III	109	23.5
	Stage IV	28	6
	**Total**	**464**	**100**
Recently diagnosed with HIV advanced disease	No	441	95
	Yes	23	5
	**Total**	**464**	**100**
	Undetectable (≤40 copies/ml)	398	85.8
Recent viral load (copies/ml)	Suppressed (> 40 copies/ml ≤ 1000copies/ml)	59	12.7
	Unsuppressed(≥ 1000copies/ml)	7	1.5
	**Total**	**464**	**100**
Have HIV/AIDS-related symptoms	No	435	93.8
	Yes	29	6.3
	**Total**	**464**	**100**
Treatment Regimen	1st line	403	86.9
	2nd line	61	13.1
	**Total**	**464**	**100**
Depressed	No	427	92
	Yes	37	8
	**Total**	**464**	**100**
Disclosed HIV/AIDS status	No	44	9.5
	Yes	420	90.5
	**Total**	**464**	**100**
Attended HIV-related counselling session	No	18	3.9
	Yes	446	96.1
	**Total**	**464**	**100**
Consume alcohol	No	170	36.6
	Yes	294	63.4
	**Total**	**464**	**100**
Smoke	No	452	97.4
	Yes	12	2.6
	**Total**	**464**	**100**

### Mean scores for global quality of life and for four domains

The mean scores for quality of life (QOL) domains were as follows: physical health score 72.93 ± (12.79), psychological health score 70.55 ± (13.35), social health score 62.03 ± (16.83), and environmental health score 64.24 ± (11.08). The overall QOL score had a mean of 269.75 ± (42.58), with values ranging from 125.89 to 386.61.

### Factors associated with the overall quality of life

Table 3 shows factors independently associated with the overall quality of life. The Overall QOL scores were significantly lower among participants from Buea Regional Hospital (B = −13.58; 95% CI: −21.02 to −6.15; p < 0.001). Marital status was positively associated with QOL, with higher scores observed among divorced (B = 24.47; 95% CI: 1.93 to 47.01; p = 0.033) and married participants (B = 11.87; 95% CI: 1.92 to 21.83; p = 0.019), compared to widowed participants. Lower education (primary level: B = −13.53; 95% CI: −25.84 to −1.22; p = 0.031) and low monthly average income (<100 USD: B = −15.31; 95% CI: −27.07 to −3.54; p = 0.011) were associated with reduced QOL. Absence of HIV-related symptoms (B = 17.40; 95% CI: 3.31 to 31.49; p = 0.015) and absence of depression (B = 25.37; 95% CI: 12.69 to 38.04; p < 0.001) were strongly associated with higher QOL scores. Alcohol non-consumption (B = −13.19; 95% CI: −20.30 to −6.08; p < 0.001) and increasing age (B = −0.85; 95% CI: −1.21 to −0.49; p < 0.001) were associated with lower overall QOL.

**Table 3:** Factors independently associated with overall quality of life scores (N = 464).

Variable	Categories	B	95% CI		p-value
			Lower	Upper	
Participants location	Buea Regional Hospital	-13.58	-21.02	-6.15	**<0.001**
	Limbe Regional Hospital	0			
Gender	Female	-6.27	-15.27	2.73	0.172
	Male	0			
Marital status	Divorced	24.47	1.93	47.01	**0.033**
	Married	11.87	1.92	21.83	**0.019**
	Single	4.55	-5.95	15.06	0.395
	Widow(er)	0			
Level of education	None	1.26	-23.16	25.69	0.919
	Primary	-13.53	-25.84	-1.22	**0.031**
	Secondary	-4.00	-15.85	7.84	0.508
	Tertiary	0			
Monthly average income(USD)	<100	-15.31	-27.07	-3.54	**0.011**
	100-200	-9.80	-21.52	1.92	0.101
	201-400	1.72	-12.56	16.00	0.813
	>400	0			
Clinical stage of HIV/AIDS	Stage I	-10.07	-25.20	5.06	0.192
	Stage II	-14.02	-30.07	2.04	0.087
	Stage III	-10.73	-26.37	4.91	0.179
	Stage IV	0			
Have HIV/AIDS related symptoms	No	17.40	3.31	31.49	**0.015**
	Yes	0			
Depressed	No	25.37	12.69	38.04	**<0.001**
	Yes	0			
Alcohol consumption	No	-13.19	-20.30	-6.08	**<0.001**
	Yes	0			
Age (Years)	Age	-0.85	-1.21	-0.49	**<0.001**

### Factors associated with the physical quality of life

Table 4 shows the factors associated with the physical health scores. Participants receiving care at Buea Regional Hospital had significantly lower physical health scores compared to those at Limbe Regional Hospital (B = −9.10; 95% CI: −11.25 to −6.95; p < 0.001). Lower income was strongly associated with reduced physical health, particularly among those earning <100 USD (B = −5.94; 95% CI: −9.13 to −2.75; p < 0.001). Clinical stage II disease was also associated with lower physical health scores (B = −5.51; 95% CI: −10.16 to −0.85; p = 0.020). Conversely, absence of HIV-related symptoms (B = 5.12; 95% CI: 1.00 to 9.24; p = 0.015) and absence of depression (B = 5.18; 95% CI: 1.51 to 8.85; p = 0.006) were associated with higher physical health scores. Alcohol non-consumption (B = −2.69; 95% CI: −4.77 to −0.62; p = 0.011) and increasing age (B = −0.35 per year; 95% CI: −0.45 to −0.26; p < 0.001) were associated with lower physical health scores.

**Table 4:** Factors independently associated with physical health scores (N = 464).

Variable	Categories	B	95% CI		p-value
			Lower	Upper	
Participants location	Buea Regional Hospital	-9.10	-11.25	-6.95	**<0.001**
	Limbe Regional Hospital	0			
Marital status	Divorced	7.43	0.90	13.97	**0.026**
	Married	1.09	-1.72	3.91	0.447
	Single	1.13	-1.92	4.18	0.466
	Widow(er)	0			
Monthly average income(USD)	<100	-5.94	-9.13	-2.75	**<0.001**
	100-200	-2.62	-5.89	0.66	0.117
	201-400	-0.77	-4.90	3.35	0.713
	>400	0			
Clinical stage of HIV/AIDS	Stage I	-3.61	-8.00	0.78	0.107
	Stage II	-5.51	-10.16	-0.85	**0.020**
	Stage III	-4.28	-8.81	0.26	0.065
	Stage IV	0			
Have HIV/AIDS related symptoms	No	5.12	1.00	9.24	**0.015**
	Yes	0			
Depressed	No	5.18	1.51	8.85	**0.006**
	Yes	0			
Alcohol consumption	No	-2.69	-4.77	-0.62	**0.011**
	Yes	0			
Age (years)	Age	-0.35	-0.45	-0.26	**<0.001**

### Factors independently associated with the psychological health scores

Participants from Buea Regional Hospital had significantly lower psychological health scores (B = −8.05; p < 0.001). Primary level education was associated with reduced psychological health compared to tertiary education (B = −4.77; p = 0.014). Absence of HIV-related symptoms (B = 5.44; p = 0.021) and absence of depression (B = 9.59; p < 0.001) were significantly associated with higher psychological health scores. Alcohol non-consumption (B = −4.83; p < 0.001) and increasing age (B = −0.16; p = 0.006) were associated with lower psychological health. This is seen in Table 5.

**Table 5:** Factors independently associated with psychological health scores (N = 464).

Variable	Categories	B	95% CI		p-value
			Lower	Upper	
Participants location	Buea Regional Hospital	-8.05	-10.54	-5.56	**<0.001**
	Limbe Regional Hospital	0			
Gender	Female	-2.27	-5.12	0.57	0.117
	Male	0			
Marital status	Divorced	6.17	-1.12	13.46	0.097
	Married	1.16	-2.08	4.39	0.483
	Single	1.48	-1.95	4.90	0.398
	Widow(er)	0			
Level of education	No formal education	-1.83	-9.60	5.93	0.644
	Primary	-4.77	-8.59	-0.96	**0.014**
	Secondary	-3.16	-6.94	0.63	0.102
	Tertiary	0			
Clinical stage of HIV/AIDS	Stage I	-2.55	-7.39	2.29	0.302
	Stage II	-3.81	-8.97	1.35	0.148
	Stage III	-3.82	-8.85	1.22	0.137
	Stage IV	0			
Recent viral load (copies/ml)	> 40 copies/ml ≤ 1000copies/ml	-2.14	-5.78	1.51	0.250
	≥ 1000copies/ml(unsuppressed)	-7.61	-16.59	1.37	0.097
	Undetectable	0			
Have HIV/AIDS related symptoms	No	5.44	0.82	10.06	**0.021**
	Yes	0			
Depressed	No	9.59	5.51	13.68	**<0.001**
	Yes	0			
Alcohol consumption	No	-4.83	-7.14	-2.51	**<0.001**
	Yes	0			
Age (Years)	Age	-0.16	-0.27	-0.05	**0.006**

### Factors independently associated with social health scores

Table 6 shows the factors independently associated with social health scores. Marital status was significantly associated with social relationship scores, with married participants having higher scores compared to widowed participants (B = 8.77; p < 0.001). Participants without depression also had significantly higher scores (B = 6.08; p = 0.022). Alcohol non-consumption (B = −3.42; p = 0.022) and increasing age (B = −0.27; p < 0.001) were associated with lower social relationship scores.

**Table 6:** Factors independently associated with social health scores (N = 464).

Variable	Categories	B	95% CI		p-value
			Lower	Upper	
Gender	Female	-3.08	-6.71	0.55	0.096
	Male	0			
Marital status	Divorced	5.94	-3.34	15.22	0.210
	Married	8.77	4.68	12.86	**<0.001**
	Single	1.38	-2.93	5.70	0.531
	Widow(er)	0			
Level of education	No formal education	1.69	-8.23	11.61	0.739
	Primary	-4.45	-9.32	0.43	0.074
	Secondary	-0.20	-5.01	4.61	0.934
	Tertiary	0			
Have HIV/AIDS related symptoms	No	5.36	-0.46	11.18	0.071
	Yes	0			
Depressed	No	6.08	0.86	11.30	**0.022**
	Yes	0			
Disclosed HIV/AIDS status	No	4.60	-0.18	9.37	0.059
	Yes	0			
Alcohol consumption	No	-3.42	-6.36	-0.49	**0.022**
	Yes	0			
Age (Years)	Age	-0.27	-0.41	-0.13	**<0.001**

### Factors independently associated with environmental health scores

Table 7 shows the factors independently associated with the environmental health scores. Participants receiving care at Buea Regional Hospital had significantly higher environmental health scores (B = 3.61; p < 0.001). Lower educational attainment (primary level: B = −4.29; p = 0.011) and lower income (<100 USD: B = −7.90; p < 0.001; 100–200 USD: B = −5.40; p = 0.001) were significantly associated with reduced environmental health scores. Absence of depression (B = 4.55; p = 0.009) was associated with higher environmental health, while alcohol non-consumption (B = −3.18; p = 0.001) and increasing age (B = −0.13; p = 0.004) were associated with lower scores.

**Table 7:** Factors independently associated with environmental health scores.

Variable	Categories	B	95% CI		p-value
			Lower	Upper	
Participants location	Buea Regional Hospital	3.61	1.62	5.59	**<0.001**
	Limbe Regional Hospital	0			
Marital status	Divorced	4.89	-1.17	10.96	0.114
	Married	0.86	-1.73	3.44	0.514
	Single	0.68	-2.11	3.47	0.632
	Widow(er)	0			
Religion	Christian	-6.25	-14.30	1.80	0.128
	Muslim	0			
Level of education	No formal education	-1.42	-7.93	5.10	0.670
	Primary	-4.29	-7.57	-1.00	**0.011**
	Secondary	-2.20	-5.36	0.96	0.172
	Tertiary	0			
Monthly average income(USD)	<100	-7.9	-10.97	-4.79	**<0.001**
	100-200	-5.4	-8.57	-2.33	**0.001**
	201-400	-1.7	-5.50	2.13	0.386
	>400	0			
Clinical stage of HIV/AIDS	Stage I	-2.99	-7.03	1.05	0.147
	Stage II	-3.99	-8.27	0.30	0.069
	Stage III	-2.22	-6.39	1.96	0.298
	Stage IV	0			
Depressed	No	4.55	1.16	7.94	**0.009**
	Yes	0			
Alcohol consumption	No	-3.18	-5.07	-1.28	**0.001**
	Yes	0			
Age (Years)	Age	-0.13	-0.22	-0.04	**0.004**

## Discussion

This study examined factors associated with quality of life (QOL) across four domains—physical, psychological, social, and environmental—as well as overall QOL among people living with HIV on antiretroviral therapy (ART). Overall, the findings suggest that QOL is multidimensional and influenced by a combination of socio-demographic, clinical, and behavioral factors.

### Socio-demographic determinants

Education and income. Lower education (primary level) and low monthly income (<100 USD) were independently associated with poorer overall, physical, and environmental health scores. These findings align with studies in Ethiopia [11,23], Pakistan [24], and China [15], where socioeconomic disadvantage predicted lower HRQOL. Socioeconomic inequities are strongly linked to HIV outcomes [25], and education may enhance health literacy, treatment adherence, and coping capacity [26,27]. The observed income gradient particularly affected physical and environmental domains, reflecting financial barriers to nutrition, housing, and healthcare access.

Married participants reported significantly higher overall and social QOL scores compared with widowed individuals. This corroborates prior findings that marital partnership and social support enhance subjective well-being and health outcomes [28,29]. Among PLWHA, social support has been consistently associated with better adherence and psychological resilience [30]. Widowed individuals may experience compounded stigma, bereavement stress, and economic vulnerability, negatively affecting QOL.

Increasing age was consistently associated with lower overall and domain-specific QOL scores. This finding is consistent with literature highlighting the “double stigma” of aging with HIV [31]. Older PLWHA often face multimorbidity, chronic pain, sleep disturbances, and social isolation [32,33] all of which can diminish physical and psychological well-being.

Participants from Buea Regional Hospital had significantly lower overall, physical, and psychological QOL scores. Contextual factors such as urban stressors, environmental management challenges, and the socio-political impact of the Anglophone crisis in the South-West Region may partially explain these disparities [34,35]. Environmental domain differences further suggest structural determinants of health, echoing WHOQOL international findings that living conditions significantly influence QOL [16].

### Clinical determinants

Absence of HIV-related symptoms was strongly associated with higher overall and domain-specific QOL scores. Although most participants were in Stage I and virally suppressed, symptomatic disease and Stage II status were linked to poorer physical QOL. These findings are consistent with [36,37], who demonstrated that clinical stability and viral suppression correlate with improved patient-reported outcomes. ART’s effectiveness in reducing symptom burden likely underpins the relatively favorable overall QOL observed.

Depression emerged as one of the strongest predictors of reduced QOL across all domains. Participants without depression had significantly higher overall (B = 25.37; p < 0.001) and domain-specific scores. This aligns with robust evidence from India, Ethiopia, and South Africa showing that depressive symptoms substantially impair HRQOL among PLWHA [9,11,38]. Mental health comorbidities may reduce ART adherence, social functioning, and perceived well-being, emphasizing the need for integrated mental health services within HIV care.

### Behavioral determinants

Interestingly, alcohol non-consumption was associated with lower QOL scores across domains. This counterintuitive finding may reflect reverse causality (i.e., individuals with poorer health abstaining from alcohol) or social-contextual factors where moderate alcohol use is embedded in social interaction. However, extensive literature demonstrates that harmful alcohol use negatively affects ART adherence and HRQOL [39–41]. The relationship between alcohol and psychological well-being is complex and often mediated by depression [42,43]. Given these mixed findings, future studies should differentiate between moderate and hazardous drinking patterns.

### Strengths and limitations of the study

This study derives its strength from the use of a standard survey instrument in assessing the quality of life of PLWHA. The adoption of higher-level statistical analytical approaches (multiple linear regression) in this study enhances the accuracy of the findings and allows for a deeper understanding of the relationships between variables. Also, a relatively large, calculated sample size was utilized to enhance the statistical power of the analysis.

Limitations of our study include its cross-sectional nature, which did not allow us to infer causality. Also, participants were recruited using a consecutive sampling approach, which may introduce selection bias and limit the generalizability of the findings to the broader population. The reliance on self-reported data introduces the possibility of social desirability bias, where participants may provide responses that align with societal expectations rather than their true experiences. An additional limitation relates to the internal consistency of some WHOQOL-BREF domains in the present study. Although the overall instrument demonstrated acceptable reliability, the social relationships domain (Cronbach’s α = 0.340) and environmental domain (Cronbach’s α = 0.575) exhibited lower internal consistency than the recommended threshold of 0.70. Therefore, associations involving these domains should be interpreted cautiously, as lower reliability may increase measurement error and reduce the precision of domain-specific estimates. While efforts were made to mitigate this bias through confidentiality and anonymity, its potential influence on the study findings should be acknowledged.

## Conclusion

In this cross-sectional study of PLWHA on ART in Buea and Limbe, overall quality of life was moderate-to-good. However, QOL remained significantly influenced by socioeconomic status, marital support, age, depression, HIV-related symptoms, alcohol use, and healthcare location. Depression and low income were the most consistent negative predictors across domains, underscoring the central role of mental health integration and socioeconomic support within HIV programs. Older age and widowhood further identified vulnerable subgroups requiring targeted psychosocial interventions. Future longitudinal research should clarify causal pathways—particularly regarding alcohol use and aging—and evaluate integrated care models that address both clinical stability and psychosocial well-being, in line with priorities highlighted in high-impact global health and mental health literature.

## Supporting information

S1 Data Quality of life Anonymous data. (XLSX)
