# Supplementary material for: Quality of life and its associated factors among adult PLWHA on ART in Fako Division, South West Region of Cameroon
Source: PLOS Ment Health. 2026 Oct 26;3(10):e0000697. doi: 10.1371/journal.pmen.0000697 (PMC13704882; doi:10.1371/journal.pmen.0000697)
Supplement: Supplementary file 1 [file pmen.0000697.s004.pdf]

Response to reviewers

Dear Reviewers,

We sincerely thank the reviewer for the careful re-evaluation of our manuscript and for the constructive comments. We acknowledge that our previous rebuttal did not provide sufficient detail regarding several methodological revisions. In response, we have substantially revised both the manuscript and the response-to-reviewers document to provide a transparent account of all changes made.

Reviewers	Comments	Response to comments
Reviewer 1	1.Sample size calculation	We have revised the description of the sample size determination in the Methods section to provide greater clarity regarding the formula, assumptions, and parameter values used. Specifically, we clarified the source of the standard deviation used in the calculation, the confidence level applied (95%), the margin of error (5%), and the adjustment for non-response. These revisions are presented in the Sampling subsection of the Methods section on page 4.<br>The sample size was determined using the single population formula $N = (Z_{\alpha/2})^2 s^2 / d^2$[10]. Where the Z-value of 1.96 for a 95% confidence level, s is the standard of deviation of 0.53 for the overall quality of life score based on previous literature, and a margin of error of 5%. A standard deviation of 0.53 was obtained from a previous study by Desta et al. (2020) conducted among people living with HIV receiving antiretroviral therapy in Ethiopia, which reported an overall health-related quality-of-life score of 3.81 ± 0.53.<br><br>This yielded an initial sample size of 432. To account for a possible non-response rate of 10%, the final sample size was adjusted to 480 participants. A total of 464 participants were recruited consecutively as they consented during follow-ups.
	2.Handling of WHOQOL-BREF scores	We confirm that WHOQOL-BREF domain scores and the overall quality-of-life score were analyzed as continuous variables. No dichotomization of WHOQOL-BREF scores was performed in the revised analysis. To improve clarity, we expanded the

		Procedures and Data Analysis sections to explicitly describe the WHOQOL-BREF scoring procedure, transformation of domain scores to a 0–100 scale, and computation of the overall QOL score as the sum of the four transformed domains. This is explained under procedures on page 5.<br><br>The scoring procedure and transformation for each domain was done using the WHO BREF and scoring instructions as seen on reference 20.<br>The overall QOL score was computed by summing the total scores of the four transformed domains as seen in a recent study on reference 23.
	3.Statistical analyses	To address the reviewer's concerns regarding analytical rigor, we revised the Data Analysis section to clearly describe the statistical procedures used.<br><br>Factors associated with overall and domain-specific quality-of-life scores were identified using multivariable linear regression models. The WHOQOL-BREF overall score and domain scores were treated as continuous dependent variables. Independent variables with $p < 0.20$ in bivariate analyses were entered into multivariable models. Variables with $p < 0.05$ in the adjusted models were considered independently associated with overall quality of life and domain-specific scores."<br>This is seen under data analysis on page 6.
	Psychometric reliability	Following the reviewer's recommendation, we added internal consistency estimates for the WHOQOL-BREF instrument and each domain. The manuscript now reports Cronbach's alpha coefficients for the overall scale and individual domains. We additionally acknowledge in the manuscript that the social relationships domain demonstrated relatively low internal consistency and discuss this as a limitation when interpreting findings from that domain as seen under strength and limitation on page 17.<br>These were the changes made under validity of the study on page 5 and 6. As seen below, "The overall quality of life scale demonstrated acceptable internal consistency (Cronbach's $\alpha = 0.783$). The physical ($\alpha = 0.719$) and psychological ($\alpha = 0.755$) domains showed

		satisfactory reliability, whereas the environmental domain (α = 0.575) was moderate and the social domain (α = 0.340) indicated poor internal consistency”
	Table duplication and presentation	All tables were carefully reviewed and reformatted. Duplicate information was removed, table numbering was corrected, and titles were revised for clarity. The presentation of regression analyses has also been standardized across all tables. This is seen on tables 3, 4, 5, 6 and 7.
	Location of revisions	The revised manuscript now contains line numbering throughout to facilitate identification of all modifications.
