# Supplementary material for: Quality of life and its associated factors among adult PLWHA on ART in Fako Division, South West Region of Cameroon
Source: PLOS Ment Health. 2026 Oct 26;3(10):e0000697. doi: 10.1371/journal.pmen.0000697 (PMC13704882; doi:10.1371/journal.pmen.0000697)
Supplement: Supplementary file 2 [file pmen.0000697.s003.docx]

Response to reviewers

Dear Reviewers,

We sincerely thank you for your thorough review and constructive feedback on our manuscript entitled:

“QUALITY OF LIFE AND ITS ASSOCIATED FACTORS AMONG ADULT PLWHA ON ART IN FAKO DIVISION, SOUTH WEST REGION OF CAMEROON”

Your insightful comments have significantly strengthened the manuscript. I have carefully revised the manuscript to address all concerns. Below is a detailed, point-by-point response indicating the changes made and the evidence supporting them.

| **Reviewers** | **Comments** | **Response** |
| --- | --- | --- |
| Reviewer one | Major concerns |  |
|  | Comment 1:Data Availability / Data transparency | The data is available. However due to ethical restrictions, the data can be requested from the Institutional Review Board of the University of Buea. |
|  | Comment 2: Inconsistent QOL instrument and conflicting description (Methods/Abstract) | We thank the reviewer for identifying this inconsistency. The study used the WHOQOL-BREF (26-item instrument). All sections of the manuscript—including the abstract, methods, results, and discussion—have been revised to consistently reflect the correct instrument. |
|  | Major Comment 3: Sample size calculation inconsistency  The sample size calculation referenced overweight/obesity prevalence rather than the study outcome. | The formula and parameters have been clarified and typographical errors corrected. |
|  | Major Comment 4: Attrition adjustment in a cross-sectional study  Attrition does not apply to cross-sectional studies | We agree with the reviewer. The wording has been revised to clarify that the adjustment was intended to account for potential non-response rather than attrition. The sampling description has been updated accordingly |
|  | Major Comment 5: Dichotomization of QOL scores  WHOQOL scores are continuous and dichotomization requires justification. | We used continuous scores |
|  | Major Comment 6: Inconsistent significance threshold  The significance threshold and variable selection strategy were inconsistently applied | The statistical analysis section has been revised to ensure methodological clarity. A consistent significance threshold of p < 0.05 is now applied throughout the manuscript. The criteria for inclusion of variables in multivariable analysis have also been clearly described. |
|  | Major Comment 7: Potential model instability  Wide confidence intervals and extreme odds ratios may reflect model instability | The data was reanalyzed |
|  | Major Comment 8: Possible duplication error in tables |  |
|  | Major Comment 9: Causal interpretations beyond study design  Statements implying causality or program effectiveness should be avoided. | We agree with the reviewer. The discussion and conclusion sections have been revised to use strictly associative language, avoiding causal interpretations. Statements suggesting program effectiveness have been removed or rephrased. |
|  | Minor Comments |  |
|  | Abstract inconsistencies | The statistical significance threshold and data collection period have been harmonized throughout the manuscript. |
|  | Administration of questionnaire: | The procedures section has been revised to clarify that the questionnaire was primarily self-administered, with assistance provided only when participants required clarification. |
|  | Psychometric reliability: |  |
|  | Terminology: | Outcome-like terms such as “achieved” have been replaced with neutral statistical descriptions (e.g., “higher scores”) |
|  | Discussion scope: | Sections speculating about contextual factors not measured in the study have been shortened and reframed as possible contextual explanations. |
|  | Editorial consistency: | Acronyms, references, tables, and formatting have been standardized throughout the manuscript. |
|  |  |  |
| Reviewer 2 | Comment 1: Inconsistent p-value thresholds | The simple linear regression was used for the bivariate analysis and the multivariate linear regression was used for the multivariate analysis. Variables with p<0.2 in the bivariate analysis entered the multivariate model. Variables with P<0.05 in the multivariate model was considered statistically significant. |
|  | Comment 2: Clarification of WHOQOL instrument | The manuscript has been revised to clearly indicate that the WHOQOL-BREF instrument (26 items) was used. All references to WHOQOL-HIV-BREF have been corrected. |
|  | Comment 3: High prevalence of good QOL | The data was reanalyzed |
|  | Comment 4: Alcohol interpretation | We appreciate this important point. The discussion has been revised to emphasize that the observed associations between alcohol consumption and certain QOL domains should not be interpreted as indicating a protective effect. |
|  | Comment 5: Regression model inconsistency | The linear regression model was used |
|  | Comment 6: Typographical corrections | This has been adjusted |
