# Supplementary material for: Quality of life and its associated factors among adult PLWHA on ART in Fako Division, South West Region of Cameroon
Source: PLOS Ment Health. 2026 Oct 26;3(10):e0000697. doi: 10.1371/journal.pmen.0000697 (PMC13704882; doi:10.1371/journal.pmen.0000697)
Supplement: Peer review history

# Peer review: Decision Letter 0

**DOI:** 10.1371/journal.pmen.0000697.r001

**Authors:** Wenwang Rao

24 Feb 2026

PMEN-D-26-00003

QUALITY OF LIFE AND ITS ASSOCIATED FACTORS AMONG ADULT PLWHA ON ART IN FAKO DIVISION, SOUTH WEST REGION OF CAMEROON

PLOS Mental Health

Dear Dr. Tambe,

Thank you for submitting your manuscript to PLOS Mental Health. After careful consideration, we feel that it has merit but does not fully meet PLOS Mental Health’s publication criteria as it currently stands. Therefore, we invite you to submit a revised version of the manuscript that addresses the points raised during the review process.

Please submit your revised manuscript by Apr 10 2026 11:59PM. If you will need more time than this to complete your revisions, please reply to this message or contact the journal office at mentalhealth@plos.org. When you're ready to submit your revision, log on to https://www.editorialmanager.com/pmen/ and select the 'Submissions Needing Revision' folder to locate your manuscript file.

Please include the following items when submitting your revised manuscript:

- A letter that responds to each point raised by the editor and reviewer(s). You should upload this letter as a separate file labeled 'Response to Reviewers'.
- A marked-up copy of your manuscript that highlights changes made to the original version. You should upload this as a separate file labeled 'Revised Manuscript with Track Changes'.
- An unmarked version of your revised paper without tracked changes. You should upload this as a separate file labeled 'Manuscript'.

Guidelines for resubmitting your figure files are available below the reviewer comments at the end of this letter.

We look forward to receiving your revised manuscript.

Kind regards,

Wenwang Rao

Academic Editor

PLOS Mental Health

Journal Requirements:

1. We noticed you have some minor occurrence of overlapping text with the following previous publication(s), which needs to be addressed:

- https://www.mdpi.com/1660-4601/20/8/5567/html

- https://doi.org/10.1371/journal.pmen.0000060

In your revision ensure you cite all your sources (including your own works), and quote or rephrase any duplicated text outside the methods section. Further consideration is dependent on these concerns being addressed.

2. Please amend your detailed Financial Disclosure statement. This is published with the article. It must therefore be completed in full sentences and contain the exact wording you wish to be published.

a. Please clarify all sources of funding (financial or material support) for your study. List the grants (with grant number) or organizations (with url) that supported your study, including funding received from your institution.

b. State the initials, alongside each funding source, of each author to receive each grant.

c. State what role the funders took in the study. If the funders had no role in your study, please state: “The funders had no role in study design, data collection and analysis, decision to publish, or preparation of the manuscript.”

d. If any authors received a salary from any of your funders, please state which authors and which funders.

If you did not receive any funding for this study, please simply state: “The authors received no specific funding for this work.”

3. In the online submission form, you indicated that "All datasets on which the conclusions of the research rely are presented in this paper. However, raw data are available from the corresponding author on reasonable request.".

All PLOS journals now require all data underlying the findings described in their manuscript to be freely available to other researchers, either

a. In a public repository,

b. Within the manuscript itself, or

c. Uploaded as supplementary information.

This policy applies to all data except where public deposition would breach compliance with the protocol approved by your research ethics board. If your data cannot be made publicly available for ethical or legal reasons (e.g., public availability would compromise patient privacy), please explain your reasons by return email and your exemption request will be escalated to the editor for approval. Your exemption request will be handled independently and will not hold up the peer review process, but will need to be resolved should your manuscript be accepted for publication. One of the Editorial team will then be in touch if there are any issues.

If the reviewer comments include a recommendation to cite specific previously published works, please review and evaluate these publications to determine whether they are relevant and should be cited. There is no requirement to cite these works unless the editor has indicated otherwise.

Additional Editor Comments (if provided):

Please carefully revise the manuscript in response to the reviewers' comments, particularly Reviewer 1's suggestion that the authors reconsider the analytical design, clarify instruments and procedures, and restructure the statistical analysis.

[Note: HTML markup is below. Please do not edit.]

Reviewers' comments:

Reviewer's Responses to Questions

**Comments to the Author**

1. Does this manuscript meet PLOS Mental Health’s publication criteria?

Reviewer #1: No

Reviewer #2: Yes

**********

2. Has the statistical analysis been performed appropriately and rigorously?

Reviewer #1: No

Reviewer #2: No

**********

3. Have the authors made all data underlying the findings in their manuscript fully available (please refer to the Data Availability Statement at the start of the manuscript PDF file)?

The PLOS Data policy

Reviewer #1: No

Reviewer #2: Yes

**********

4. Is the manuscript presented in an intelligible fashion and written in standard English?

Reviewer #1: No

Reviewer #2: Yes

**********

Reviewer #1: Thank you for the opportunity to review this manuscript. The topic is highly relevant to public health and mental health in populations living with HIV, particularly because it addresses quality of life (QOL) within a specific regional context. The study has the potential to contribute to the local literature and to inform service improvements. However, in its current form, the work presents important methodological and statistical inconsistencies that compromise the validity of the findings, the interpretability of the reported associations, and adherence to transparency and reproducibility guidelines. I recommend that the authors reconsider the analytical design, clarify instruments and procedures, and restructure the statistical analysis before a potential resubmission.

Major Concerns

1) Data Availability / Data transparency (PLOS Data Policy)

Excerpt in question: “However, raw data are available from the corresponding author on reasonable request.”

Issue: The “available upon request” formulation generally does not meet the requirement of making all data underlying the findings fully and unrestrictedly available. In addition, the manuscript relies heavily on analytical decisions (e.g., QOL categorization) that require greater transparency regarding both data and code.

Recommendation: Deposit the anonymized dataset in an appropriate repository and provide the analysis code/script. If genuine ethical restrictions apply (e.g., risk of re-identification), provide a detailed justification and propose an alternative controlled-access mechanism through an institutional repository.

2) Inconsistent QOL instrument and conflicting description (Methods/Abstract)

Excerpts in question:

Abstract: “Quality of life was assessed using … WHOQOL-HIV-BREF.”

Methods/Variables/Procedures: “This variable was determined using the WHOQOL BREF tool.” and “The WHOQOL-BREF instrument comprises 26 items…”

Issue: WHOQOL-HIV-BREF and WHOQOL-BREF are different instruments; this inconsistency compromises validity, comparability, and interpretation (including domains and items).

Recommendation: Clearly correct which instrument was applied, justify the choice, describe the version/language/local validation, and align the Abstract, Methods, Results, and Discussion accordingly. Include internal consistency metrics (e.g., Cronbach’s alpha by domain) and evidence of cultural/linguistic adaptation.

3) Inadequate sample size calculation and logic inconsistent with the outcome (Sampling)

Excerpt in question: “sample size … Cauhran formula with an estimated prevalence of key overweight/obesity (63%)”

Issue: The sample size calculation was based on the prevalence of overweight/obesity (???), rather than the primary outcome (QOL) or parameters related to score variability.

Recommendation: Recalculate/justify sample size based on QOL (expected mean/standard deviation, minimally clinically important difference, or prevalence of low QOL if dichotomization is unavoidable—although not recommended).

4) “Attrition” adjustment in a cross-sectional study (Sampling)

Excerpt in question: “An additional (35%) participants were included to account for potential attrition…”

Issue: In a cross-sectional study, “attrition” (loss to follow-up) does not apply.

Recommendation: Fully revise this section; if non-response or refusals occurred, report refusal rates and reasons, and consider potential selection bias.

5) Dichotomization (“Good/Poor” and “achieved”) without a defined cutoff or justification (Results/Analysis/Discussion)

Excerpts in question:

Results/Figure 1: “Good” vs “Poor” categories.

Text: “had achieved physical… psychological… social… environmental domain”

Abstract: “96.3% reported a good overall quality of life”

Issue: WHOQOL produces continuous scores; dichotomization reduces information, may inflate odds ratios, and can generate ceiling effects (as suggested by the very high percentages). No cutoff definition or theoretical/empirical rationale is provided.

Recommendation: Report and analyze continuous domain scores (and, if appropriate, use linear/generalized models with assumption checks). If the authors insist on dichotomization, they must explicitly define and justify the cutoff (based on literature/validation); otherwise, the “good/poor” proportions are not interpretable.

6) Inconsistency in significance threshold and variable selection (Methods vs Results)

Excerpts in question:

Methods: “p-value < 0.02 were eligible for multivariate analysis… Statistical significance … less than 0.02”

Results: multiple findings with p=0.021; p=0.023; p=0.026; p=0.030 treated as significant.

Issue: The significance criterion changes/is applied inconsistently, undermining the credibility of the analytical pipeline.

Recommendation: Standardize and justify the threshold (e.g., p<0.05, with discussion of multiple comparisons when relevant), declare the strategy a priori, and apply it consistently. Clearly specify which variables enter the model and why.

7) Potentially unstable models (rare events), extreme ORs, and very wide CIs

Excerpts in question:

Example: “AOR: 12.282; CI: 3.49–43.22” (Table 7)

Example: “AOR: 5.004; CI: 1.27–19.69” (Table 3)

Issue: With overall QOL classified as “good” for 96.3% of participants, there are likely very few “events” (small n in the “poor” group). This violates events-per-variable requirements and promotes overfitting and inflated ORs, producing very wide CIs.

Recommendation: Redesign the analytical strategy: avoid dichotomization; reduce the number of predictors; use penalized approaches (e.g., Firth) if logistic regression is retained; report model diagnostics and EPV; provide cell counts for key categories.

8) Inconsistency/potential error in tables (duplicated values and coding)

Excerpt in question (Table 5):

“Disclosed … No 0.356 (0.19–0.69)”

“Consume alcohol No 0.356 (0.19–0.69)”

Issue: Identical AOR and CI for two distinct variables is unlikely and suggests a typographical or copy-paste error.

Recommendation: Audit tables, rerun analyses, provide output/model specification, and correct inconsistencies.

9) Causal overreach and conclusions beyond the study design (Discussion/Conclusion)

Excerpts in question:

“affirming the transformative impact of ART…”

“suggesting effective HIV care delivery…”

“These findings support the effectiveness of current ART programs…”

Issue: A hospital-based cross-sectional study does not allow causal inference or evaluation of “program effectiveness.” Moreover, consecutive recruitment at refill visits likely overrepresents adherent, clinically stable patients, inflating QOL estimates.

Recommendation: Rewrite the Discussion/Conclusion using strictly associative language; include selection bias and limited generalizability as central limitations; avoid strong programmatic recommendations without direct evidence.

Minor concerns

A) Abstract – redundancies and inconsistencies

Excerpts in question:

“Statistical significance was set at p value<0.05…” (Abstract) vs Methods p<0.02

Data collection period: “from June 2024 to July 2024” vs Methods “June 1 to August 31, 2024”

Recommendation: Harmonize the period and significance threshold; reduce generic statements in the abstract; include the regression type and an operational definition of “good QOL.”

B) Methods – operational coherence (self-administered vs interview)

Excerpt in question: “WHOQOL-BREF is a self-administered questionnaire…” and then “…consented … was interviewed.”

Recommendation: Clarify administration mode (self-administered vs interviewer-administered), assistant training, and potential biases (social desirability/interviewer effects).

C) Psychometric validity/reliability

Excerpt in question: focus only on “face validity” and “content validity.”

Recommendation: Report internal consistency (Cronbach’s alpha by domain) and discuss cultural/linguistic validation of the instrument in the local context.

D) Terminology and scientific writing (“achieved”, “good outcomes”)

Excerpts in question: repeated use of “achieved” and “good outcomes” for QOL domains.

Recommendation: Replace with score-consistent language (“higher scores”, “mean domain score”) and avoid outcome-like terms that suggest causality.

E) Discussion with unmeasured hypotheses

Excerpts in question: extensive explanations about security, infrastructure, displacement, industrialization, etc., without direct measures in the study.

Recommendation: Keep these as hypotheses/context, explicitly stating they were not measured; avoid deterministic post-hoc storytelling.

F) Editorial consistency (formatting, minor issues, standardization)

Recommendation: Standardize acronyms (PLWHA/PLWHIV), check currency units (USD/XAF), verify references, and align tables/figures with the main text.

G) Interpretive coherence regarding alcohol

Excerpts in question: repeated association suggesting better QOL among alcohol consumers, with occasionally contradictory explanations.

Recommendation: Treat this as likely confounding/residual confounding or a socioeconomic proxy; avoid any interpretation implying benefit from alcohol; suggest additional analyses (stratification/robust adjustment) or a more cautious discussion.

Reviewer #2: In the abstract section, P value<0.05 but in methodology it is mentioned that P<0.001/p=0.001. I am not sure about it which is correct or how correctly the statistical measure was done. Please review this and make sure it is consistent with the analysis.

WHOQOL-BREF and WHOQOL-HIV BREF are two different tools. WHOQOL-HIV BREF contains 31 items and includes spirituality/religion domain , whereas WHOQOL-BREF contains 26 items. Please clarify which tool was actually used.

The reported prevalence of good overall QOL (96.3%) is unusually high compared to similar studies in sub-Saharan Africa, and the criteria used to classify “good” versus “poor” QOL should be described as per my point of view. The interpretation of alcohol consumption as being associated with better psychological, social, and environmental QOL should be handled cautiously to avoid implying a protective effect. Additionally, the manuscript states that multiple linear regression was used, yet adjusted odds ratios are presented, suggesting logistic regression; this is one of the major discrepancy which needs to be addressed.

In the sampling section, you have written "Cauhran Formula" but I assume that it is "Cochran Formula"

In table 6, Buea Regional Hospital spells wrong, correct that too.

However, I would recommend you to go through the statistical analysis again and verify it. Otherwise, the paper has potential to be published.

**********

what does this mean?). If published, this will include your full peer review and any attached files.

**Do you want your identity to be public for this peer review?**  If you choose “no”, your identity will remain anonymous but your review may still be made public.

For information about this choice, including consent withdrawal, please see our Privacy Policy

Reviewer #1: No

Reviewer #2: **Yes:**  Rehnuma Abdullah

**********

[NOTE: If reviewer comments were submitted as an attachment file, they will be attached to this email and accessible via the submission site. Please log into your account, locate the manuscript record, and check for the action link "View Attachments". If this link does not appear, there are no attachment files.]

# Author comment: Author response to Decision Letter 1

**DOI:** 10.1371/journal.pmen.0000697.r002

21 Apr 2026

Attachment Submitted filename: Response to reviewers.docx

# Peer review: Decision Letter 1

**DOI:** 10.1371/journal.pmen.0000697.r003

**Authors:** Wenwang Rao

3 Jun 2026

PMEN-D-26-00003R1

QUALITY OF LIFE AND ITS ASSOCIATED FACTORS AMONG ADULT PLWHA ON ART IN FAKO DIVISION, SOUTH WEST REGION OF CAMEROON

PLOS Mental Health

Dear Dr. Tambe,

Thank you for submitting your manuscript to PLOS Mental Health. After careful consideration, we feel that it has merit but does not fully meet PLOS Mental Health’s publication criteria as it currently stands. Therefore, we invite you to submit a revised version of the manuscript that addresses the points raised during the review process.

Please submit your revised manuscript by Jul 03 2026 11:59PM. If you will need more time than this to complete your revisions, please reply to this message or contact the journal office at mentalhealth@plos.org. When you're ready to submit your revision, log on to https://www.editorialmanager.com/pmen/ and select the 'Submissions Needing Revision' folder to locate your manuscript file.

Please include the following items when submitting your revised manuscript:

- A letter that responds to each point raised by the editor and reviewer(s). You should upload this letter as a separate file labeled 'Response to Reviewers'.
- A marked-up copy of your manuscript that highlights changes made to the original version. You should upload this as a separate file labeled 'Revised Manuscript with Track Changes'.
- An unmarked version of your revised paper without tracked changes. You should upload this as a separate file labeled 'Manuscript'.

Guidelines for resubmitting your figure files are available below the reviewer comments at the end of this letter.

As the corresponding author, your ORCID iD is verified in the submission system and will appear in the published article. PLOS supports the use of ORCID, and we encourage all coauthors to register for an ORCID iD and use it as well. Please encourage your coauthors to verify their ORCID iD within the submission system before final acceptance, as unverified ORCID iDs will not appear in the published article. *Only*  the individual author can complete the verification step; PLOS staff *cannot*  verify ORCID iDs on behalf of authors.

We look forward to receiving your revised manuscript.

Kind regards,

Wenwang Rao

Academic Editor

PLOS Mental Health

Journal Requirements:

If the reviewer comments include a recommendation to cite specific previously published works, please review and evaluate these publications to determine whether they are relevant and should be cited. There is no requirement to cite these works unless the editor has indicated otherwise.

Please review your reference list to ensure that it is complete and correct. If you have cited papers that have been retracted, please include the rationale for doing so in the manuscript text, or remove these references and replace them with relevant current references. Any changes to the reference list should be mentioned in the rebuttal letter that accompanies your revised manuscript. If you need to cite a retracted article, indicate the article’s retracted status in the References list and also include a citation and full reference for the retraction notice.

Additional Editor Comments (if provided):

Please carefully respond to Reviewer 1’s comments and provide a specific point-by-point reply according to the reviewer’s requirements, with particular attention to the statistical analysis.

[Note: HTML markup is below. Please do not edit.]

Reviewers' comments:

Reviewer's Responses to Questions

**Comments to the Author**

Reviewer #1: (No Response)

Reviewer #2: All comments have been addressed

**********

publication criteria?

Reviewer #1: Partly

Reviewer #2: (No Response)

**********

3. Has the statistical analysis been performed appropriately and rigorously?

Reviewer #1: No

Reviewer #2: I don't know

**********

4. Have the authors made all data underlying the findings in their manuscript fully available (please refer to the Data Availability Statement at the start of the manuscript PDF file)?

The PLOS Data policy

Reviewer #1: Yes

Reviewer #2: Yes

**********

5. Is the manuscript presented in an intelligible fashion and written in standard English?

Reviewer #1: Yes

Reviewer #2: Yes

**********

Reviewer #1: I appreciate the effort made to respond to the reviewers’ comments. I hope this feedback will be received as a constructive contribution to clarify and strengthen the points that remain pending in the manuscript.

The authors have addressed some comments in principle. However, several responses remain insufficiently detailed in the response-to-reviewers document. Important methodological issues have not yet been adequately demonstrated as resolved in the rebuttal, particularly regarding the sample size calculation, the handling and possible dichotomization of WHOQOL scores, table duplication, and psychometric reliability. In addition, some responses remain vague, such as “the data were reanalyzed” or “this has been adjusted,” without clearly specifying what was changed, where the change was made in the manuscript, or how the revised analyses address the concerns raised by the reviewers. This is particularly relevant for the statistical analysis, as the response does not provide sufficient detail to determine whether the revised analyses were performed appropriately and rigorously. I recommend that the authors provide a revised and detailed point-by-point response, including specific page and line references, as well as updated tables and analyses where applicable, before the manuscript can be considered for further evaluation. I also suggest that the manuscript lines be numbered to facilitate the identification of the changes made.

Reviewer #2: I believe it is eligible for submission now

**********

what does this mean?). If published, this will include your full peer review and any attached files.

**Do you want your identity to be public for this peer review?**  If you choose “no”, your identity will remain anonymous but your review may still be made public.

For information about this choice, including consent withdrawal, please see our Privacy Policy

Reviewer #1: No

Reviewer #2: **Yes:**  Rehnuma Abdullah

**********

[NOTE: If reviewer comments were submitted as an attachment file, they will be attached to this email and accessible via the submission site. Please log into your account, locate the manuscript record, and check for the action link "View Attachments". If this link does not appear, there are no attachment files.]

# Author comment: Author response to Decision Letter 2

**DOI:** 10.1371/journal.pmen.0000697.r004

27 Jun 2026

Attachment Submitted filename: Response to Reviewers.pdf

# Editor report: Decision Letter 2

**DOI:** 10.1371/journal.pmen.0000697.r005

**Authors:** Wenwang Rao

4 Aug 2026

QUALITY OF LIFE AND ITS ASSOCIATED FACTORS AMONG ADULT PLWHA ON ART IN FAKO DIVISION, SOUTH WEST REGION OF CAMEROON

PMEN-D-26-00003R2

Dear Dr Tambe,

We are pleased to inform you that your manuscript 'QUALITY OF LIFE AND ITS ASSOCIATED FACTORS AMONG ADULT PLWHA ON ART IN FAKO DIVISION, SOUTH WEST REGION OF CAMEROON' has been provisionally accepted for publication in PLOS Mental Health.

Before your manuscript can be formally accepted you will need to complete some formatting changes, which you will receive in a follow up email. A member of our team will be in touch with a set of requests.

Please note that your manuscript will not be scheduled for publication until you have made the required changes, so a swift response is appreciated.

IMPORTANT: The editorial review process is now complete. PLOS will only permit corrections to spelling, formatting or significant scientific errors from this point onwards. Requests for major changes, or any which affect the scientific understanding of your work, will cause delays to the publication date of your manuscript.

If your institution or institutions have a press office, please notify them about your upcoming paper to help maximize its impact. If they'll be preparing press materials, please inform our press team as soon as possible -- no later than 48 hours after receiving the formal acceptance. Your manuscript will remain under strict press embargo until 2 pm Eastern Time on the date of publication. For more information, please contact mentalhealth@plos.org.

Thank you again for supporting Open Access publishing; we are looking forward to publishing your work in PLOS Mental Health.

Best regards,

Wenwang Rao

Academic Editor

PLOS Mental Health

***********************************************************

Reviewer Comments (if any, and for reference):
